# Organ‐Specific Dedifferentiation and Epigenetic Remodeling in In Vivo Reprogramming

**DOI:** 10.1111/acel.70268

**Published:** 2025-10-20

**Authors:** Beom‐Ki Jo, Seung‐Yeon Lee, Hee‐Ji Eom, Jumee Kim, Hyuk‐Jin Cha

**Affiliations:** ^1^ College of Pharmacy, Seoul National University Seoul Republic of Korea; ^2^ College of Pharmacy, Sookmyung Women's University Seoul Republic of Korea; ^3^ Drug Information Research Institute, Sookmyung Women's University Seoul Republic of Korea; ^4^ Research Institute of Pharmaceutical Sciences, Seoul National University Seoul Republic of Korea

**Keywords:** epigenetic reprogramming, in vivo reprogramming, injury induced reprogramming, rejuvenation, tissue regeneration, transient regenerative progenitors, Yamanaka factors

## Abstract

The advent of in vivo reprogramming through transient expression of the Yamanaka factors (OCT4, SOX2, KLF4, and c‐MYC) holds strong promise for regenerative medicine, despite ongoing concerns about safety and clinical applicability. This review synthesizes recent advances in in vivo reprogramming, focusing on its potential to restore regenerative competence and promote rejuvenation across diverse tissues, including the retina, skeletal muscle, heart, liver, brain, and intestine. We highlight mechanistic parallels and distinctions between injury‐induced dedifferentiation and OSKM‐mediated reprogramming, emphasizing the roles of dedifferentiation, transient regenerative progenitors, and epigenetic remodeling. Critical safety considerations—such as teratoma formation, organ failure, and loss of cell identity—are discussed alongside strategies designed to mitigate these risks, like cyclic induction and targeted delivery. Finally, we briefly note the growing translational interest in this field, alongside directing readers to recent reviews for detailed coverage of biotech initiatives. Collectively, this work underscores the transformative potential of in vivo reprogramming for both tissue regeneration and rejuvenation, while stressing the importance of precise spatiotemporal control for its safe clinical application.

## Graphical abstract

Transient in vivo expression of Yamanaka factors (OSKM) enhances regenerative competence and rejuvenation across multiple tissues. We highlight mechanistic links between injury‐induced dedifferentiation and OSKM reprogramming, while emphasizing safety challenges and the need for precise spatiotemporal control to enable clinical translation.

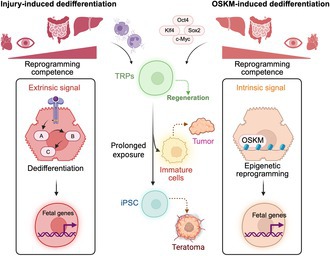

## 1 Introduction

**FIGURE 1:**
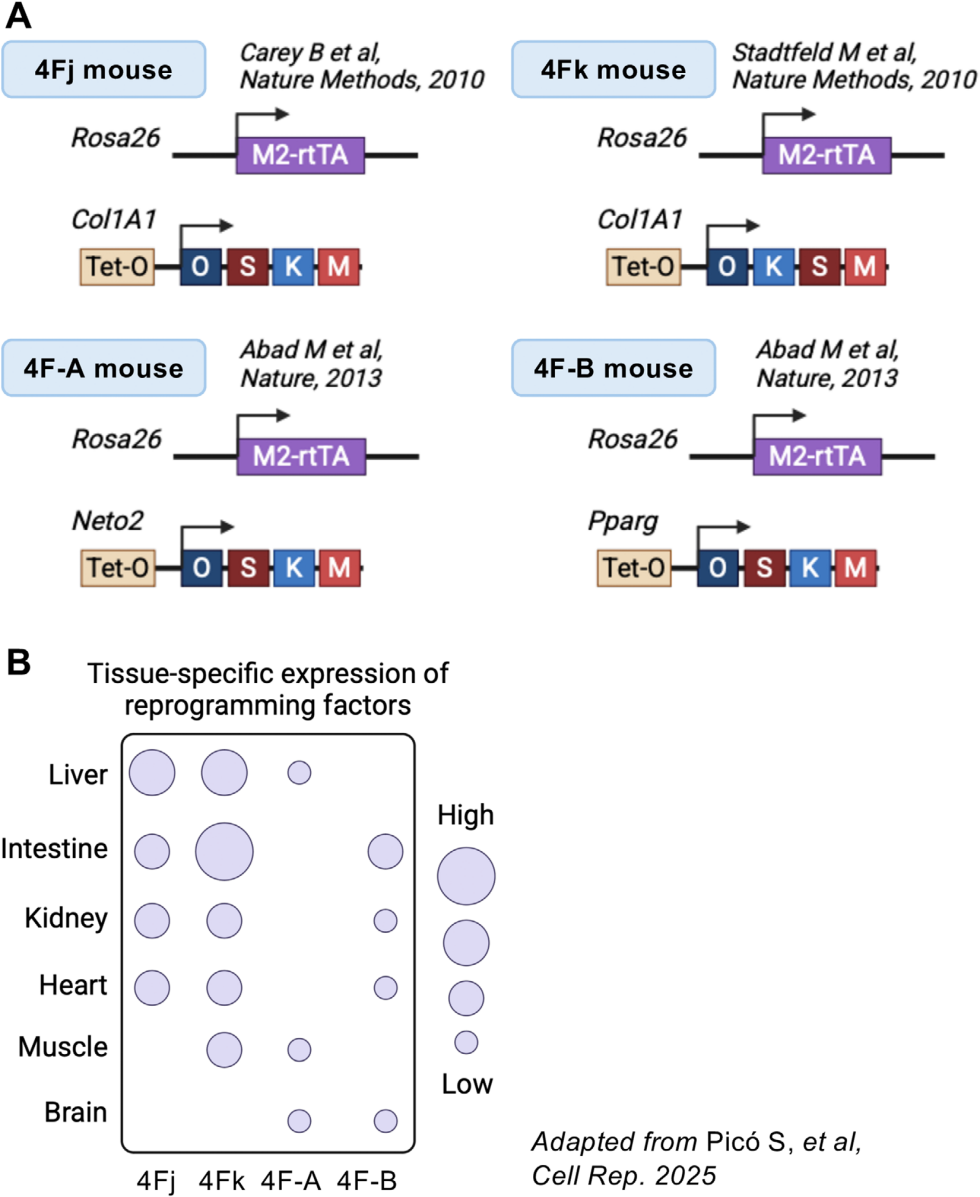
Comparison of various transgenic mouse models expressing OSKM. (A) Schematic representation of four different OSKM (Oct4, Sox2, Klf4, and c‐Myc) inducible mouse models used for in vivo reprogramming studies. (B) Schematic representation of reprogramming factor expression levels across organs and strains. Bubble size indicates the relative expression of reprogramming factors, with larger bubbles signifying higher expression levels. This comparative analysis provides insights into strain‐specific differences in reprogramming potential across multiple tissues.

Reprogramming through expression of the Yamanaka factors (OCT4, SOX2, KLF4, and c‐MYC, collectively OSKM) is one of the main breakthroughs in stem cell research, enabling not only the production of patient‐specific pluripotent stem cells for cell therapy and disease modeling (Yamanaka 2020) but also the control of cell fate determination (Kim et al. 2011; Margariti et al. 2012). In addition to cellular reprogramming in vitro, in vivo reprogramming with OSKM has been achieved using inducible transgenic mouse models. These models employ a Tet‐O promoter system to control the expression of OSKM, allowing precise temporal regulation of reprogramming through doxycycline (Dox) administration. Two well‐established models, 4Fj and 4Fk, respectively express OSKM or OKSM cassettes inserted at the *Col1a1* locus (Carey et al. 2010; Stadtfeld et al. 2010). The alternative models 4F‐A (or 4FsA) and 4F‐B (or 4FsB) similarly feature OSKM cassettes respectively integrated at the *Neto2* and *Pparg* loci (Abad et al. 2013) (Figure 1A). The transient OSKM induction enabled by the Tet‐O system provides a platform for studying the dynamic effects of reprogramming across tissues and organs, with the degree of reprogramming varying depending on organ and experimental context (Pico et al. 2025). That is, the chromatin landscape and promoter accessibility vary across organs, and hence OSKM expression patterns in 4F mice show striking tissue dependence, with robust induction in the intestine, liver, and skin, and comparatively lower activation in the brain, heart, and skeletal muscle (Pico et al. 2025). This tissue specificity is briefly summarized in Figure 1B.

**FIGURE 2:**
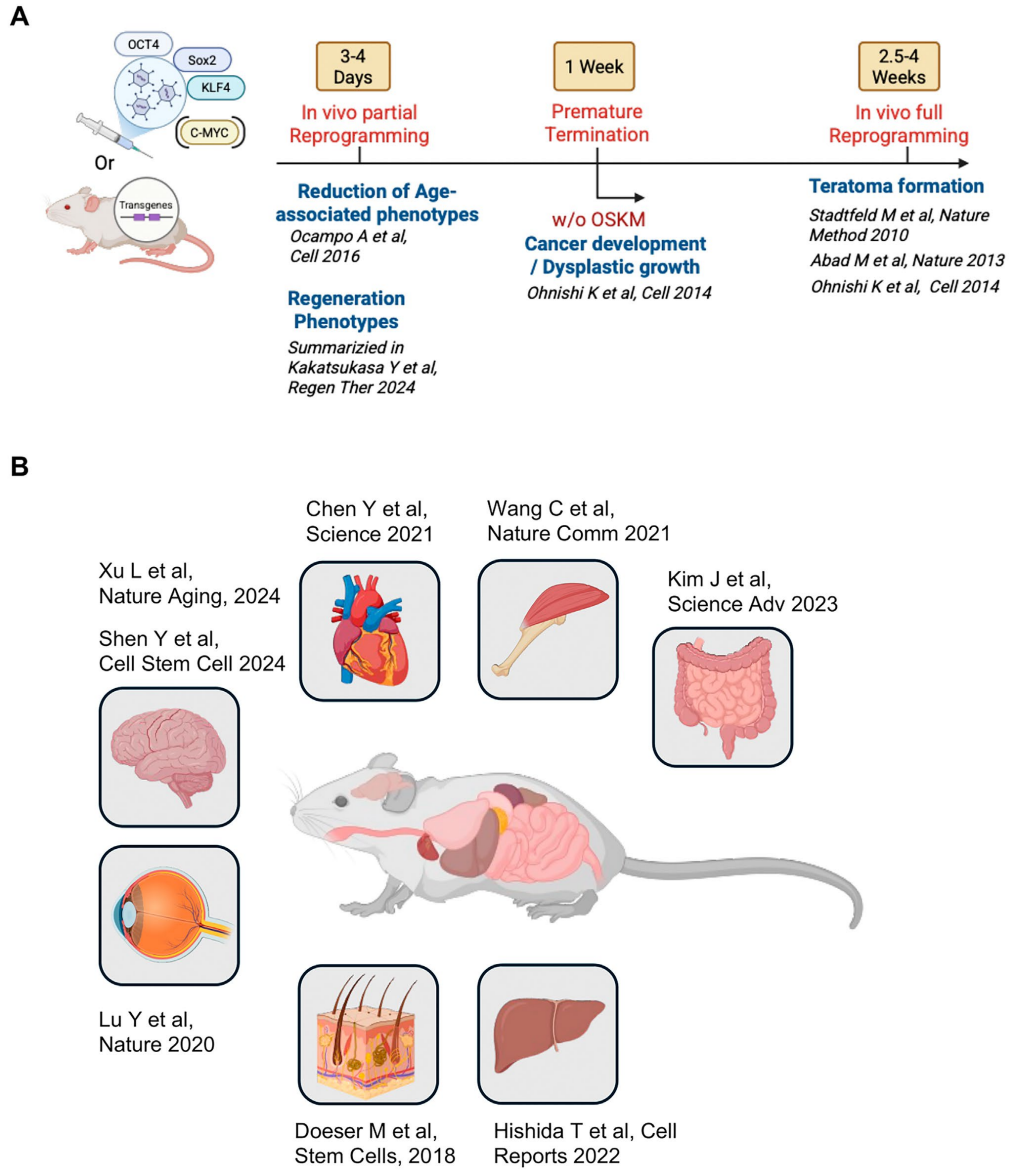
Outcomes of in vivo reprogramming using OSKM. (A) In vivo full reprogramming leads to teratoma formation, a hallmark of pluripotency. Prematurely terminated OSKM expression results in dysplastic or cancerous growth, highlighting the tumorigenic risks associated with incomplete reprogramming. In contrast, in vivo partial OSKM reprogramming offers two distinct therapeutic benefits: (1) reduction of age‐associated phenotypes, contributing to systemic rejuvenation, and (2) induction of regenerative phenotypes, which enhance tissue repair and regeneration without teratoma formation. This highlights the therapeutic potential of OSKM reprogramming while emphasizing the need to balance its benefits against the risks associated with full reprogramming. (B) Schematic of in vivo reprogramming through transient expression of OSK(M), which induces regeneration phenotypes in various organs and has broad potential for tissue repair and rejuvenation. The highlighted organs include the brain, heart, skeletal muscle, eyes, skin, liver, and intestines. Each box emphasizes the specific tissues in which regeneration has successfully been demonstrated, further supporting its potential for clinical applications in regenerative medicine and aging‐related therapies.

Continuous induction of OSKM over weeks has been shown to produce teratomas in multiple organs (Abad et al. 2013; Stadtfeld et al. 2010). Indeed, even transient induction—such as 7 days of OSKM expression followed by Dox withdrawal—can initiate dysplastic changes and tumor formation, with neoplasms developing by 2 to 3 weeks post‐induction in organs such as the pancreas, liver, and kidney. However, when injected into other blastocysts, the reprogrammed cells successfully develop into chimeras with “normal‐looking tissue” (Ohnishi et al. 2014). This implies that re‐differentiated cells which have undergone reprogramming, such as dysplasia or loss of identity, can retain their ability to contribute to normal tissue development when placed in a supportive environment. The timeline and outcomes of OSKM expression are summarized in Figure 2A.

A pivotal in vivo study by Ocampo et al. explored the potential of partial reprogramming through transient induction of OSKM (hereafter “OSKM reprogramming”) to mitigate aging‐related phenotypes and extend lifespan using a progeria mouse model that harbored mutant lamin A, the protein responsible for human Hutchinson‐Gilford progeria syndrome (HGPS). Cyclic induction of OSKM using the Dox‐inducible system (e.g., 2 days ON and 5 days OFF, repeated weekly) significantly extended lifespan and improved multiple aging‐related histological and functional phenotypes such as spine curvature, skin integrity, and cardiovascular function, without teratoma formation (Ocampo et al. 2016). In addition, the results revealed enhanced regenerative capacity in physiologically aged mice, including improved pancreatic function and muscle regeneration following injury; the concomitant restoration of epigenetic markers like H3K9me3 suggested that OSKM reprogramming resets the epigenetic landscape to counteract the progressive age‐related loss of heterochromatin and DNA methylation fidelity (Ocampo et al. 2016). Since the publication of that groundbreaking study in 2016, and particularly from 2020 onward, several key papers have further demonstrated partial OSKM, OSK (OCT4, SOX2, and KLF4), or OSKMLN (OSKM, LIN28, and NANOG) induction to have regenerative effects in vivo (Chen et al. 2021; Hishida et al. 2022; Kim et al. 2023; Lu et al. 2020; Wang et al. 2021) and to promote rejuvenation capacity both in vivo in mouse models (Browder et al. 2022; Rodriguez‐Matellan et al. 2020) and in vitro in human cell models (Gill et al. 2022; Olova et al. 2019; Roux et al. 2022; Sarkar et al. 2020). While numerous review articles have extensively summarized the research on rejuvenation and the remodeling of age‐associated epigenetic marks (Cipriano et al. 2024; Paine et al. 2024; Puri and Wagner 2023; Sichani et al. 2024; Yucel and Gladyshev 2024; Zhang et al. 2020), this review takes a unique perspective by emphasizing how OSKM or OSK‐ reprogramming contributes to organ‐specific regeneration, particularly the parallels and differences between natural, injury‐induced dedifferentiation and OSKM reprogramming. By offering a detailed analysis of regenerative processes and their therapeutic implications, this review provides a complementary yet distinct addition to the existing body of literature.

## 2 Main text

Several interesting studies have examined the effect of OSKM reprogramming in cancer. One demonstrated that transient OSKM expression in *Kras* mutant mice alters the epigenetic profile of differentiated acinar cells and induces poorly‐differentiated cancers (Shibata et al. 2018), suggesting that OSKM expression drives cancer development through epigenetic changes associated with dedifferentiation. Meanwhile, in a mouse model harboring the *MLL‐AF9* fusion gene, which is associated with acute myeloid leukemia (Somervaille and Cleary 2006), induction of OSKM leads to selective eradication of leukemia cells while having minimal impact on normal hematopoietic cells (Wang, Lu, et al. 2019). The observed leukemia cell death was linked to increased chromatin accessibility near genes encoding apoptotic regulators and to downregulation of the H3K9me3 histone mark, which is associated with chromatin compaction and gene repression. This linking was validated through inhibition of KDM3A, the H3K9 demethylase, which partially rescued leukemia cells from apoptosis (Wang, Lu, et al. 2019). Finally, a particularly insightful study by Ohnishi et al. (2014) demonstrated that iPSCs derived from dysplastic kidney tumors, formed following premature OSKM termination, produce normal, non‐neoplastic tissues when injected into blastocysts to create chimeric embryos. This regenerative outcome was associated with epigenetic reprogramming, including erasure of tumor‐associated transcriptional and chromatin abnormalities. Together, these studies underscore the pivotal role of epigenetic landscape remodeling in determining the fate of OSKM reprogramming—whether toward malignancy, cell death, or regeneration. Unlike tumor cells, where OSKM may exacerbate instability, in vivo reprogramming of normal somatic cells seeks to reverse age‐ or injury‐associated epigenetic modifications, thereby re‐establishing plasticity through a controlled and context‐dependent reset.

## 3 Tissue Regeneration

Beyond the transformative potential of epigenetic remodeling observed in cancer (Shibata et al. 2018; Somervaille and Cleary 2006; Wang, Lu, et al. 2019), in vivo reprogramming of normal cells can restore cellular plasticity and promote tissue regeneration across multiple organs, as summarized in Figure 2B. Following the landmark study that demonstrated OSKM reprogramming as a strategy to reverse aging phenotypes (Ocampo et al. 2016), subsequent work began to explore its regenerative effects in diverse tissue contexts. In this section, we focus on the application of OSKM‐mediated reprogramming in tissue repair, highlighting how transient OSKM induction drives epigenetic remodeling that restores cellular function and enhances regenerative capacity in damaged tissues (Figure 2B). One of the earliest demonstrations came from a skin injury model using 4Fj mice, wherein Doeser et al. (2018) showed that OSKM induction reduced fibrotic responses and inhibited fibroblast “trans‐differentiation” into myofibroblasts; this highlighted the ability of OSKM to modulate cellular identity and promote tissue repair, albeit with delayed wound closure. Since then, numerous studies have expanded the investigation of OSKM reprogramming's regenerative effects across a variety of organs, revealing its potential to rejuvenate aged tissues and enhance cellular plasticity.

Although OSKM can broadly promote regeneration, the regenerative context differs fundamentally from organ to organ. Some tissues—such as the retina, skeletal muscle, and heart—possess only limited or no intrinsic regenerative capacity. In these settings, OSKM reprogramming functions as an exogenous driver, overcoming epigenetic barriers to induce repair. By contrast, organs such as the liver and intestine retain innate regenerative potential, largely through injury‐induced dedifferentiation of mature cells into progenitor‐like states. In these cases, OSKM reprogramming amplifies or parallels endogenous plasticity programs rather than introducing regeneration *de novo*. To reflect this distinction, the following sections are organized into two parts: (1) In vivo Partial Reprogramming to Unlock Restricted Regeneration and (2) In vivo Partial Reprogramming to Enhance Natural Dedifferentiation Programs.

### 3.1 In Vivo Partial Reprogramming to Unlock Restricted Regeneration

#### 3.1.1 Retina Regeneration by OSK Through DNA Demethylation

The eye is uniquely suited for localized gene therapy, leading to adeno‐associated virus (AAV) vectors being the method of choice for treating ocular diseases. Specifically, the eye's enclosed anatomy, immune‐privileged environment, and direct accessibility allow precise delivery via AAV to targeted retinal cell types with minimal systemic exposure (He et al. 2023). In adult mice, intravitreal injection of AAV encoding OSK—excluding c‐Myc to minimize oncogenic risk (Nakagawa et al. 2008)—restores the regenerative capacity of retinal ganglion cells (RGCs) (Lu et al. 2020), which normally lose the ability to regrow axons shortly after birth (Goldberg et al. 2002). In models of optic nerve crush (Lu et al. 2020) and glaucoma (Karg et al. 2023), OSK expression enhances RGC survival, promotes robust axon regeneration over millimeter‐scale distances, and enables sustained recovery of visual function, which benefits persist for months. In addition, long‐term continuous expression for over 20 months causes no detectable retinal structural abnormalities or tumor formation (Karg et al. 2023). Mechanistically, OSK reprogramming in the retina counteracts injury‐induced epigenetic alterations, including accelerated DNA methylation aging, and restores youthful methylation patterns at genes associated with neuronal function (Lu et al. 2020). Indeed, the regeneration mediated by OSK requires TET2‐dependent DNA demethylation, as disruption of *Tet2* completely abolishes OSK‐induced RGC survival and axon regrowth. Given the role of TET2 in producing 5‐hydroxymethylcytosine via cytosine oxidation, active DNA demethylation has emerged as indispensable for full OSK‐mediated recovery (Lu et al. 2020).

In parallel, the injured retina can also initiate regeneration through Müller glia (MG), the principal retinal macroglia, which reenter the cell cycle and transiently acquire progenitor‐like properties. That is, retinal injury activates a transcriptional program associated with glial reactivity and neurogenic priming (Hoang et al. 2020), which is accompanied by chromatin remodeling that increases accessibility at cis‐regulatory elements linked to progenitor identity. In regenerative species such as zebrafish and chick, injury‐induced DNA demethylation at neurogenic gene loci facilitates the transition of MG into progenitor cells (Luz‐Madrigal et al. 2020; Powell et al. 2013). In mammals, however, DNA methylation is maintained after injury by DNMT3A and DNMT3B, restricting MG reprogramming. Consistently, regenerating RGCs demonstrate differential methylation at the *Dnmt3a* and *Dnmt3b* loci (Rizk et al. 2023), and inhibition of *Dnmt3a* in mice promotes optic nerve regeneration and vision recovery (Tai et al. 2023). Much like TET2‐dependent demethylation is indispensable for OSK‐induced regeneration of RGCs, relieving the repression imposed by DNMT3A‐mediated methylation in MG appears critical for their neurogenic conversion. Collectively, these findings identify DNA methylation as a unifying regulatory axis across both neuronal and glial regeneration that determines whether retinal cells can surmount intrinsic regenerative barriers and restore visual function. Precise modulation of DNA methylation—whether by promoting or removing methyl marks—thus emerges as a decisive factor in enabling retinal regeneration.

#### 3.1.2 Cardiac Regeneration

Unlike the bone marrow and gastrointestinal tract, which maintain homeostasis through continuous self‐renewal from resident stem cells, the fully developed heart remains a prototypical non‐regenerative organ in most mammals, with only rare exceptions. That is, whereas neonatal mammals, along with lower vertebrates such as zebrafish and newts, can regenerate cardiac tissue, adult mammalian hearts do not have sufficient regenerative capacity to restore function after injury (Garbern and Lee 2022). Recent evidence indicates the adult heart to consist primarily of fully differentiated cells and to lack tissue‐specific stem cells (Kretzschmar et al. 2018; Li, He, et al. 2018). While early reports proposed the existence of cardiac stem cells in adults (Bearzi et al. 2007), this has been largely discredited following multiple retractions of key studies (Kaiser 2018). The adult liver is also widely accepted as lacking resident stem cells for tissue homeostasis (Grompe 2014), yet exhibits remarkable regenerative capacity through active reprogramming of hepatocytes (Miyajima et al. 2014; Yanger et al. 2014). In contrast, adult cardiomyocytes are highly resistant to reprogramming (Porrello et al. 2011).

The regenerative capacity of the neonatal heart is associated with profound changes in transcription, metabolism, and the epigenome (Puente et al. 2014; Quaife‐Ryan et al. 2017), which are induced by a unique immune response (Miyajima et al. 2014; Wang, Cui, et al. 2019). Interestingly, transient expression of OSKM in the adult heart induces a fetal‐like transcriptional program characterized by the dedifferentiation of cardiomyocytes, including sarcomeric disassembly, re‐expression of developmental markers (e.g., α‐SMA and GATA4), and cell‐cycle re‐entry. The dedifferentiated cells regain proliferative potential, enabling them to contribute to regeneration. Functionally, this leads to improved cardiac output, reduced fibrosis, and increased survival after myocardial infarction compared to untreated controls, underscoring dedifferentiation through OSKM induction as the primary regenerative mechanism in the adult heart (Chen et al. 2021; Yao and Wang 2020; Zhu et al. 2021). Further supportive evidence comes from both zebrafish (Jopling et al. 2010; Kikuchi et al. 2010) and mammalian models, where dedifferentiation precedes regenerative responses. Importantly, in vivo cardiac reprogramming requires intensified OSKM expression (Chen et al. 2021)—that is, administration of a dose five times higher than that used for full reprogramming (Haenebalcke et al. 2013)—but prolonged expression (e.g., 7 weeks) in cardiomyocytes also leads to tumor formation. These tumors express Nanog, a marker of full reprogramming, and can generate mouse chimeras (Chen et al. 2021). Similarly, *Myh6*‐promoter–driven OSKM induction in cardiomyocytes for 18 days results in teratoma formation with expression of *Nanog* and endogenous *Pou5f1*, providing clear evidence of progression from dedifferentiation toward pluripotency (de Lázaro et al. 2021). These findings underscore the necessity of tightly controlling OSKM expression in order to achieve safe regenerative outcomes. At the same time, targeted OSKM delivery to cardiomyocytes by AAV for up to 1 month was found to induce a limited set of reprogramming‐related genes without *Nanog* expression or tumorigenesis, but also failed to improve cardiac regeneration after injury (Kisby et al. 2021), underscoring the low reprogramming competence of adult cardiomyocytes.

#### 3.1.3 Skeletal Muscle Regeneration

After continuous OSKM induction over weeks, 4F‐A and 4F‐B mice develop teratomas, an event representative of full reprogramming (Abad et al. 2013). Similar to the senescence‐induced reprogramming observed during whole‐body regeneration in hydra (Salinas‐Saavedra et al. 2023), the cellular reprogramming that leads to teratoma formation in mice is facilitated in the setting of injury or age due to secretion of interleukin‐6 (IL‐6), which occurs as a senescence‐associated secretory phenotype (SASP) (Mosteiro et al. 2016) dependent on p16Ink4a (Mosteiro et al. 2018), a cell cycle‐dependent kinase inhibitor and well‐established marker of aging. That is, cells expressing p16Ink4a (encoded by *Cdkn2a*) often undergo cellular senescence following tissue damage and contribute to the local microenvironment by secreting SASP factors, which include cytokines, growth factors, and proteases; these factors then influence neighboring cells and tissues by promoting inflammation, altering extracellular matrix composition, and modulating tissue regeneration and repair processes (Muñoz‐Espín and Serrano 2014). The augmenting effect of IL‐6 on in vivo reprogramming by OSKM has been reproduced in skeletal muscle, with both aged and injured muscles demonstrating greater susceptibility to the development of teratomas derived from reprogramming of PAX7‐positive (PAX7+) satellite cells (Chiche et al. 2017).

Notably, PAX7+ satellite cells, which are indispensable for skeletal muscle regeneration (Sambasivan et al. 2011), generally exist in a quiescent state until being activated upon injury, which process is tightly controlled by Polycomb‐repressive complex 2 (PRC2)‐dependent histone modification (Juan et al. 2011). The core histone methyltransferase of PRC2, EZH2, is highly expressed in embryonic stem cells (Lee, Li, et al. 2022) and has critical roles in maintaining pluripotency (Collinson et al. 2016) and facilitating OSKM‐mediated reprogramming (Fragola et al. 2013); correspondingly, its repression of cell‐cycle inhibitor genes such as *CDKN1A* (encoding p21^CIP1^) and *CDKN1C* (encoding p57^KIP2^), as has been reported in cancer cells (Fan et al. 2011; Yang et al. 2009), may promote re‐entry of PAX7^+^ satellite cells into the cell cycle during OSKM induction. This possibility has been examined by the Belmonte group; specifically, Wang et al. (2021) demonstrated that short‐term expression of OSKM in myofibers enhances the abundance of PAX7+ satellite cells by repressing secretion of WNT4, a protein that maintains satellite cell quiescence via repressing YAP (Eliazer et al. 2019). This enhancement leads to reduced scar formation and improves functional recovery of muscle after injury. Importantly, OSKM expression has only marginal direct effect on PAX7+ satellite cells, suggesting that control of the surrounding microenvironment is critical for the transition of these and other stem cells from quiescent to active state. In this vein, cyclic degradation of *Wnt4* mRNA by CRISPR‐RfxCas13 (CasRx), a class 2 type VI CRISPR‐Cas RNA endonuclease that induces degradation of a target RNA (Konermann et al. 2018), has been shown to effectively promote muscle regeneration (Wang et al. 2021) and may represent a feasible therapeutic approach.

### 3.2 In Vivo Partial Reprogramming to Enhance Natural Dedifferentiation Programs

#### 3.2.1 Liver Regeneration

##### 3.2.1.1 Injury‐Induced Dedifferentiation

The liver is unique among mammalian organs for its remarkable regenerative capacity (Michalopoulos and Bhushan 2021). Under homeostatic conditions, hepatocytes are largely quiescent—exhibiting low turnover and long lifespans—but upon injury, they can rapidly reenter the cell cycle, with proliferation occurring broadly across the hepatic lobule (Huppert and Schwartz 2023; Michalopoulos and Bhushan 2021; Sun et al. 2020). Zone 2 hepatocytes, situated between the periportal (Zone 1) and pericentral (Zone 3) regions, display the highest proliferative activity and disproportionately sustain hepatocyte maintenance during homeostasis (He et al. 2021; Wei et al. 2021). Thus, liver homeostasis is maintained primarily by unidirectional self‐renewal of mature hepatocytes, without reliance on a classical stem cell hierarchy.

Following acute or chronic injury, this paradigm shifts. When hepatocyte proliferation is impaired or massive cell loss occurs, hepatocytes undergo dedifferentiation, acquiring progenitor‐like features that enable re‐entry into the cell cycle and tissue regeneration (Li et al. 2023; Tanimizu et al. 2014; Tarlow et al. 2014); in particular, hepatic progenitor cells transiently arise with upregulated expression of fetal or progenitor markers such as *Afp*, *Sox9*, and *Krt19*, contributing to parenchymal restoration where canonical proliferation is insufficient (Lu et al. 2015). This process represents not passive regression but an actively regulated state transition, characterized by transcriptional reprogramming and chromatin remodeling (Li et al. 2019; Macchi and Sadler 2020). Ultimately, the plasticity is underpinned by epigenetic remodeling, with chromatin regulators such as *Arid1a*, *Uhrf1*, and the PRC2 complex controlling hepatocyte competency for dedifferentiation and proliferation (Aloia 2021). Loss of *Arid1a* enhances chromatin accessibility at YAP/TEAD targets (Li et al. 2019); notably, YAP is a central regulator that drives dedifferentiation in both hepatocytes and cholangiocytes (Planas‐Paz et al. 2019; Yimlamai et al. 2014). Depletion of *Uhrf1* similarly induces global DNA hypomethylation that is balanced by a redistribution of H3K27me3, relieving repression at pro‐regenerative loci (Wang, Zhang, et al. 2019). Injury also reactivates fetal gene programs, consistent with a recapitulation of embryonic transcriptional states (Ben‐Moshe et al. 2022; Li et al. 2023). Collectively, these findings highlight liver injury‐induced dedifferentiation as a form of adaptive reprogramming, orchestrated by chromatin dynamics to enable robust regeneration. Moreover, they underscore that hepatocytes possess intrinsically high reprogramming competence, a term originally introduced to describe the pre‐open chromatin state that permits hepatocyte‐to‐progenitor conversion (Li et al. 2020). More broadly, we use this concept to denote the innate capacity of a given cell type to undergo dedifferentiation and epigenetic remodeling in response to reprogramming cues.

##### 3.2.1.2 OSKM‐Induced Dedifferentiation

Building on the above‐described endogenous mechanisms, in vivo reprogramming has been leveraged to experimentally induce dedifferentiation and enhance hepatic regeneration. A pivotal study by Hishida et al. (2022) showed that transient hepatocyte‐specific OSKM expression for just 1 day downregulated hepatocyte identity genes such as *Hnf4α* while also transiently activating fetal genes such as *Afp*, producing a cell population similar to that observed after injury‐induced dedifferentiation (Nakano et al. 2017). ATAC‐seq analysis further revealed OSKM induction to lead to (i) global chromatin remodeling, (ii) opening of pluripotency‐associated motifs, (iii) erosion of hepatocyte enhancer accessibility, and (iv) establishment of a poised, plastic chromatin state. Importantly, this partial reprogramming, though insufficient to induce pluripotency given that markers such as *Nanog* and *Rex1* were not detectably expressed, generates a distinct hepatocyte subpopulation expressing epigenetic modifiers including *Ezh2*, *Tet1*, and *Dnmt1* (Hishida et al. 2022). These changes were reversible upon OSKM withdrawal, and no tumorigenesis was observed up to 9 months post‐induction. Mechanistically, Topoisomerase 2a (*Top2a*) has emerged as a key mediator of OSKM‐driven dedifferentiation, facilitating mitotic chromatin remodeling that is essential for both reprogramming (Hishida et al. 2022) and developmental regulation (Miller et al. 2017; Thakurela et al. 2013).

Notably, unlike injury‐induced dedifferentiation, which requires immune‐derived cytokine signaling to trigger hepatocyte plasticity (Li et al. 2023), in vivo OSKM reprogramming operates largely in an immune‐independent manner (our unpublished data and personal communication). Hence, OSKM‐induced dedifferentiation faithfully recapitulates core features of injury‐induced plasticity—including reactivation of fetal gene programs, genome‐wide chromatin reconfiguration, and relief of epigenetic repression—while bypassing the requirement for inflammatory cues, providing a tunable, immune‐independent strategy for unlocking regenerative potential in severely injured livers.

#### 3.2.2 Intestine Regeneration

##### 3.2.2.1 Injury‐Induced Dedifferentiation

**FIGURE 3:**
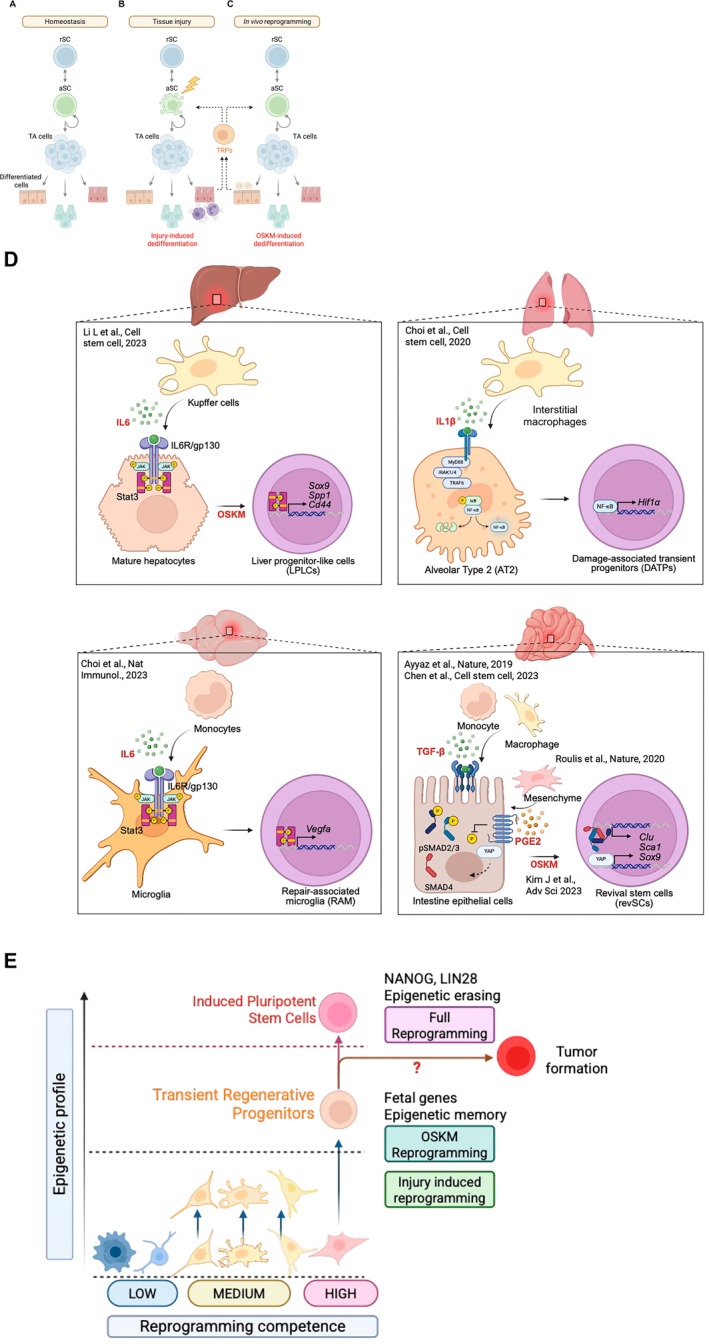
Cellular dynamics during tissue regeneration upon injury or induction by in vivo reprogramming. Illustration of cellular dynamics under three different conditions: (A) homeostasis, (B) tissue injury, and (C) in vivo reprogramming. This figure highlights the distinct cellular responses and regenerative potential under injury‐induced repair and OSKM reprogramming. (A) Under homeostasis, reserve stem cells (RSCs) remain in a quiescent state, serving as a reservoir to maintain the stem cell pool. They transition into active stem cells (aSCs) to support routine cell turnover and tissue maintenance. aSCs undergo asymmetric self‐renewal to generate transit‐amplifying (TA) cells, which rapidly proliferate and further differentiate into fully matured cells that sustain tissue function. (B) Upon tissue injury, aSCs are damaged and/or eliminated, triggering a repair response. A tissue‐specific cell type with high reprogramming competence then undergoes injury‐induced dedifferentiation, generating transient regenerative progenitors (TRPs) that support tissue recovery and regeneration. (C) In the context of in vivo reprogramming, cellular plasticity is enhanced, leading to the formation of transient regenerative progenitors that further contribute to tissue regeneration. Through this process, OSKM‐induced dedifferentiation enables cells to revert to a fetal‐like state, facilitating regeneration beyond natural repair mechanisms. This figure highlights the distinct cellular responses and regenerative potential under injury‐induced repair and OSKM reprogramming, emphasizing their respective roles in tissue regeneration. (D) Schematic illustration of the potential for injury‐induced dedifferentiation and OSKM reprogramming across organ systems in a mouse model. Highlighted tissues, including the liver, lung, brain, and small intestine represent organs where injury‐induced dedifferentiation has been observed. (Top left) In the liver, hepatic injury activates resident Kupffer cells to secrete IL‐6, which induces STAT3 phosphorylation in neighboring hepatocytes. This activation promotes hepatocyte dedifferentiation toward liver progenitor‐like cells (LPLCs), characterized by Sox9, Cd44, and Spp1 expression, while suppressing Hnf4α. (Li et al. 2023) Hepatocyte dedifferentiation also has been observed by partial reprogramming without inflammatory response. (Hishida et al. 2022) (Top right) In the lung, bleomycin‐induced injury leads to the infiltration of interstitial macrophages that secrete IL‐1β. This cytokine activates NF‐κB signaling in alveolar type II (AT2) epithelial cells via the MyD88–IRAK4–TRAF6 cascade, upregulating Hif1a and promoting their transition into damage‐associated transient progenitors (DATPs). (Choi et al. 2020) (Bottom left) After cerebrovascular injury, infiltrating monocytes secrete IL‐6 which activates the JAK/STAT3 signaling cascade in resident microglia. This pathway drives the transcription of Vegfa, a potent proangiogenic factor, thereby programming microglia into repair‐associated microglia (RAMs) (Choi et al. 2023) (Bottom right). In the intestine, injury activates cytokine signaling pathways, including TGF‐β and PGE2, which induce dedifferentiation of intestinal epithelial cells into revival stem cells (revSCs), marked by Sca1, Clu, and Sox9 expression. (Ayyaz et al. 2019; Chen et al. 2023; Roulis et al. 2020) These cell states are transient and associated with fetal‐like reprogramming regulated by YAP and canonical TGF‐β signaling. Notably, OSKM induction alone is sufficient to generate revSC‐like populations in the absence of injury, by activating epithelial Ptgs1‐dependent PGE2 synthesis and the downstream EP4–YAP axis. (Kim et al. 2023) (E) Somatic cell types across the body display variable levels of reprogramming competence. Cells with high competence (e.g., intestinal epithelial cells and hepatocytes) are more susceptible to substantial epigenetic alteration upon OSKM reprogramming and injury‐induced reprogramming, leading to dedifferentiation into transient regenerative progenitors. The phenotypic properties of these progenitors vary among organs, likely reflecting differences in epigenetic memory retained by the corresponding mature cells. Prolonged OSKM expression drives further induction toward pluripotent stem cells characterized by *Nanog* and *Lin28* expression, although this process is also associated with tumorigenic risks such as teratoma formation. Collectively, the distinct outcomes of OSKM versus injury‐induced reprogramming are determined by intrinsic reprogramming competence as well as the duration of exposure to reprogramming stimuli.

The intestinal epithelium is renewed by active intestinal stem cells (aISCs), also referred as crypt base columnar cells, which are positive for leucine‐rich repeat containing G protein‐coupled receptor 5 (LGR5); these cells generate absorptive and secretory progenitors for all epithelial lineages during normal homeostasis (van der Flier and Clevers 2009) (Figure 3A). Severe injury (e.g., radiation or chemotherapy) ablates proliferative aISCs and transit‐amplifying cells, causing crypt erosion (Beumer and Clevers 2016). Classically, regeneration was attributed to activation of quiescent “+4” reserve stem cells expressing *Bmi1*, *mTert*, *Lrig1*, and *HopX* (Beumer and Clevers 2016), which are resistant to DNA damage (Yan et al. 2012). Alternatively, injury‐triggered induction of cellular plasticity or cellular reprogramming—also termed adaptive cellular reprogramming (Jessen et al. 2015) or injury‐induced dedifferentiation (Higa et al. 2022)—occurs in differentiated cells or progenitors, leading to the formation of a transient population of injury‐responsive cells (Kim et al. 2023; Lee, Kim, et al. 2022) that serves to compensate for the loss of aISCs (Figure 3B).

The extensive evidence compiled in this review underscores the remarkable plasticity of intestinal cells and their ability to transiently dedifferentiate to compensate for stem cell loss. Lineage‐tracing studies have provided direct evidence that a number of cell types play key roles in intestinal regeneration, including Prox1+ enteroendocrine cells (Yan et al. 2017), Paneth cells (Yu et al. 2018), Dclk1+ tuft cells (Westphalen et al. 2014), Bmi1+ enteroendocrine progenitors (Jadhav et al. 2017), CD69+/CD274+ goblet precursors (Jadhav et al. 2017), Alpi+ absorptive progenitors (Tetteh et al. 2016), Atoh1+ (Tomic et al. 2018), or Dll1+ secretory precursors (van Es et al. 2012), and bipotential precursors with both absorptive and secretory potential (Murata et al. 2020). Following cryptic damage by ionizing radiation, injury‐induced dedifferentiation produces a clusterin (*Clu*)‐expressing cell population, referred to as “revival stem cells (revSCs)”, that are extremely rare under homeostatic conditions; these cells can engender all the major intestinal cell types in a process that involves a fetal gene program (“fetal‐like reversion”) (Viragova et al. 2024) and is dependent on YAP (Ayyaz et al. 2019; Qu et al. 2021) and p53 (Morral et al. 2024). YAP activation is tightly controlled by microenvironmental cues, particularly those that suppress Hippo signaling following injury; correspondingly, extracellular matrix (ECM) remodeling provides important upstream inputs (Yui et al. 2018). Soluble mediators also contribute, such as TGFβ1 (Chen et al. 2023); prostaglandin E2 (PGE2) secreted by rare pericryptal Ptgs2+ fibroblasts (Roulis et al. 2020), monocytes (Li, Soendergaard, et al. 2018), and macrophages (Meriwether et al. 2022); immunoglobulin superfamily containing leucine‐rich repeat (ISLR), a secreted stromal protein (Xu et al. 2020); and macrophage‐derived factors such as neuregulin 1 (NRG1) and osteopontin (OPN) (Moraitis et al. 2025). Ultimately, both stromal and immune‐derived signals converge on YAP to drive fetal conversion and robust intestinal regeneration.

##### 3.2.2.2 OSKM‐Induced Dedifferentiation

The ability of diverse epithelial cell types to dedifferentiate upon injury suggests they possess reprogramming competence (Li et al. 2020)—the intrinsic capacity to remodel chromatin and acquire stem‐like potential in response to reprogramming cues. In turn, OSKM induction in epithelial cells should phenocopy injury‐induced dedifferentiation (Figure 3C). Indeed, Kim et al. showed that short‐term OSKM expression increases the plasticity of enterocytes and tuft, goblet, and Paneth cells, producing two populations: revSC‐like cells (Ayyaz et al. 2019) localized to crypts and villus‐specific atrophy‐induced villus epithelial cells (aVECs) that promote villus recovery (Ohara et al. 2022). The distinct spatial origins of these populations point to there being multiple “cells of origin” with reprogramming competence (Kim et al. 2023), and both populations exhibit high YAP activity and fetal protein expression, consistent with injury‐induced programs.

A key mechanistic insight is that OSKM reprogramming activates the PGE2–YAP axis cell‐autonomously, without requirement for immune input. Transcriptomic analysis of intestinal organoids—devoid of niche‐derived signals—along with subsequent biochemical studies has revealed a striking induction of *Ptgs1*, which encodes cyclooxygenase 1 (COX1), a key enzyme in PGE2 biosynthesis that is essential for gastrointestinal mucosal integrity (Sigthorsson et al. 2002). The related gene *Ptgs2*, which encodes cyclooxygenase 2 (COX2), is in contrast drastically induced following acute injury and mediates inflammatory responses, making it a key target for anti‐inflammatory therapies (Flower 2003). Pharmacological inhibition of COX1, but not COX2, effectively suppresses PGE2 production upon OSKM induction, leading to repression of YAP activation, fetal‐like transitions, and the formation of revSC‐like cells, ultimately impairing regenerative capacity (Kim et al. 2023).

Notably, PGE2‐mediated YAP activation via PGE2 receptor 4 (EP4) has been well established as a key driver of colon regeneration (Kim et al. 2017). More broadly, PGE2 has a conserved role in regeneration across mammalian tissues, including the intestine (Miyoshi et al. 2017; Roulis et al. 2020), kidney (Chen et al. 2021), skeletal muscle (Ho et al. 2017; Palla et al. 2021), and other organ systems (Zhang et al. 2015); it is also important in gecko tail regeneration (Xu et al. 2020)—as extensively reviewed in (Cheng et al. 2021)—and hence its function in tissue repair appears to be an evolutionarily conserved mechanism. This is further supported by the conservation of COX pathway proteins across *Euteleostomi* (i.e., bony vertebrates) (Wei et al. 2023), reinforcing the fundamental role of this pathway in regeneration (Goessling et al. 2009). Ultimately, it is clear that PGE2‐mediated YAP activation serves as a shared mechanism between injury‐induced dedifferentiation and OSKM reprogramming for intestinal regeneration, but an open question remains as to whether this conserved mechanism also operates in other OSKM‐induced regenerative contexts, such as the liver, skeletal muscle, and retina. The phenotypic outcomes of OSKM reprogramming are summarized in Table 1.

**TABLE 1:** Summary of OSKM reprogramming studies across various tissues and organs. This table presents an overview of in vivo reprogramming studies conducted in organs, focusing on the mouse models, reprogramming factors, induction methods, key phenotypes, and mechanisms.

Organ	Mouse model	Factors	Induction approach	Key phenotypes	Mechanism	References
Retina	N.A.	OSK	AAV	Regeneration of retinal ganglion cells Vision restoration Reversal of DNA methylation age	TET2‐dependent DNA demethylation	Lu et al. (2020)
Brain	4FA‐NES 5xFAD	OSKM	SYN1‐dependent rtTA‐expressing AAV, Dox‐inducible neural stem/progenitor‐specific expression (Nestin‐Cre; Col1a1‐TetO‐OSKM; Rosa26‐rtTA)	Increased proliferation and cortical expansion Improved behavioral performance Prevented cognitive declines and ameliorated hippocampal plaques	N.D.	Shen et al. (2024)
	4Fj	OSKM	Dox‐inducible systemic expression (Col1a1‐TetO‐OSKM; Rosa26‐rtTA)	Increased neuroblast proportion to youthful levels in the subventricular zone neurogenic niche Enhanced neurogenesis and restored progenitor pools Partial reversal of age‐associated transcriptomic changes Improved cell adhesion and chromatin remodeling in progenitors	N.D.	Xu et al. (2024)
Heart	4F^Heart^	OSKM	Dox‐inducible cardiomyocyte‐specific expression (Xmlc2‐Cre; Col1a1‐TetO‐OSKM; Rosa26‐rtTA)	Transition of adult cardiomyocytes to fetal‐state cardiomyocytes Reentry of cardiomyocytes to mitosis Heart regeneration after injury	N.D.	Chen et al. (2021)
Skeletal muscle	Acta‐Cre/4F^het^	OSKM	Dox‐inducible myofiber‐specific expression (Acta1‐Cre; Col1a1‐TetO‐OSKM; Rosa26‐rtTA)	Promoted satellite cell proliferation in myofibers Promoted reprogramming by injury	Inhibition of *Wnt4* expression through p21	Wang et al. (2021)
Liver	Hep‐4F	OSKM	Dox‐inducible hepatocyte‐specific expression (Alb‐Cre; Col1a1‐TetO‐OSKM; Rosa26‐LSL‐rtTA‐GFP)	Transition of adult hepatocytes to a progenitor state Increased cell proliferation Changes in DNA accessibility Enhanced liver regenerative capacity	Epigenetic modification by TOP2a	Hishida et al. (2022)
Intestine	4Fk	OSKM	Dox‐inducible systemic OSKM expression (Col1a1‐TetO‐OSKM; Rosa26‐rtTA)	Intestine dedifferentiation Formation of two types of “injury responsive cells” (revSCs and aVECs) Enhanced intestine regeneration by IR	*Ptgs1* induction and PGE2 production YAP activation	Kim et al. (2023)
Skin	4Fj	OSKM	Dox‐inducible systemic OSKM expression (Col1a1‐TetO‐OSKM; Rosa26‐rtTA)	Suppressed fibroblast‐to‐myofibroblast trans‐differentiation Reduced scar tissue formation in vivo	Diminished TGF‐β signaling	Doeser et al. (2018)
Peripheral nerve	4Fj	OSKM	Aged iOSKM mice (Col1a1‐TetO‐OSKM; Rosa26‐rtTA; 20 months old, pulsed 2d ON/5d OFF), Schwann cell–specific iOSKM (Plp1‐CreERT2; Col1a1‐TetO‐OSKM; Rosa26‐rtTA)	Restored Schwann cell plasticity and repair phenotype Reduced pathological accumulation of Runx2^+^ transitional Schwann cells Re‐established stress granule homeostasis Enhanced axonal regeneration and remyelination Improved functional recovery in aged mice	Restoration of stress granules homeostasis via eIF2α phosphorylation and autophagy‐mediated clearance	Wang, Wang, et al. (2025)

Abbreviations: N.A., not applicable; N.D., not determined.

### 3.3 Cell‐Extrinsic Mechanisms of Transient Regenerative Progenitor Formation in Injury‐Induced Dedifferentiation

The phenomenon of injury‐induced dedifferentiation, in which transient progenitor‐like populations are produced that support regeneration, is not confined to the intestine but is observed across multiple organs. In the brain, ischemic injury induces repair‐associated microglia (RAM) (Mastorakos et al. 2021); in the lung, alveolar type 2 cells give rise to damage‐associated transient progenitors (DATPs) (Choi et al. 2020); in the kidney, proximal tubule epithelial cells dedifferentiate into renal proximal tubular epithelial cells (PTECs) (Chang‐Panesso et al. 2019); and in the liver, hepatocytes and cholangiocytes generate diverse progenitor‐like states, including liver progenitor‐like cells (LPLCs) and interface hepatocytes (Ben‐Moshe et al. 2022; Li et al. 2023). In the intestine, multiple populations emerge under stress, such as revSCs (Ayyaz et al. 2019) and injury‐responsive cells (Kim et al. 2023; Lee, Kim, et al. 2022). Although the nomenclature and origin of these various populations differ, their transiently plastic cell states all share the conserved function of compensating for stem cell loss. To unify these observations, we refer to them here as transient regenerative progenitors (TRPs)—cells arising from dedifferentiation that acquire stem‐like properties and contribute to tissue restoration.

A unifying theme is that TRP induction depends on cell‐extrinsic signals from the damaged microenvironment. Acute injury provokes inflammation and ECM remodeling (Lane et al. 2014), which together activate signaling cascades such as YAP/TAZ, JAK/STAT3, and TGFβ–SMAD, thereby licensing differentiated cells to re‐enter a plastic state. YAP activation, central to fetal‐like conversion, is driven by ECM remodeling (Moya and Halder 2019; Yui et al. 2018) and reinforced by soluble mediators such as IL‐6, secreted by Kupffer cells in the liver and by monocytes in the brain (Choi et al. 2023; Li et al. 2023); IL‐1β from interstitial macrophages in the lung (Choi et al. 2020); TGFβ1 from monocytes and macrophages (Chen et al. 2023); PGE2 from pericryptal Ptgs2^+^ fibroblasts, monocytes, and macrophages (Li, Soendergaard, et al. 2018; Meriwether et al. 2022; Roulis et al. 2020); and neuregulin‐1/osteopontin from macrophages (Moraitis et al. 2025). These signals converge on epithelial or parenchymal target cells—hepatocytes, microglia, alveolar epithelial cells, and the intestinal epithelium—driving their dedifferentiation into TRPs that transiently regenerate tissue (Figure 3D). Thus, while the precise effector cell type varies, the extrinsic nature of the trigger is consistent: cytokine and stromal cues reprogram mature cells into regenerative intermediates.

### 3.4 Cell‐Intrinsic Mechanisms of Transient Regenerative Progenitor Formation in OSKM‐Induced Dedifferentiation

Based on findings in the intestinal epithelium (Kim et al. 2023) and emerging evidence in the liver (unpublished observations), it is plausible that OSKM‐induced dedifferentiation in other organs follows a similar trajectory to injury‐induced dedifferentiation, as outlined in Figure 3. Especially, OSKM reprogramming activates similar regenerative pathways in a cell‐intrinsic manner, without requiring inflammatory cytokines or niche remodeling. Evidence from the intestinal epithelium demonstrates that OSKM directly induces *Ptgs1* (COX1), which leads to PGE2 production and YAP activation and thereby drives revSC‐like cell formation even in organoids devoid of stromal support (Kim et al. 2023). Pharmacological inhibition of COX1, but not COX2, abolishes this response, distinguishing OSKM from injury, which relies on inflammation‐driven COX2 induction (Flower 2003). Hence, OSKM autonomously mobilizes a pathway—PGE2–EP4–YAP—that is normally contingent on immune‐derived signals.

**FIGURE 4:**
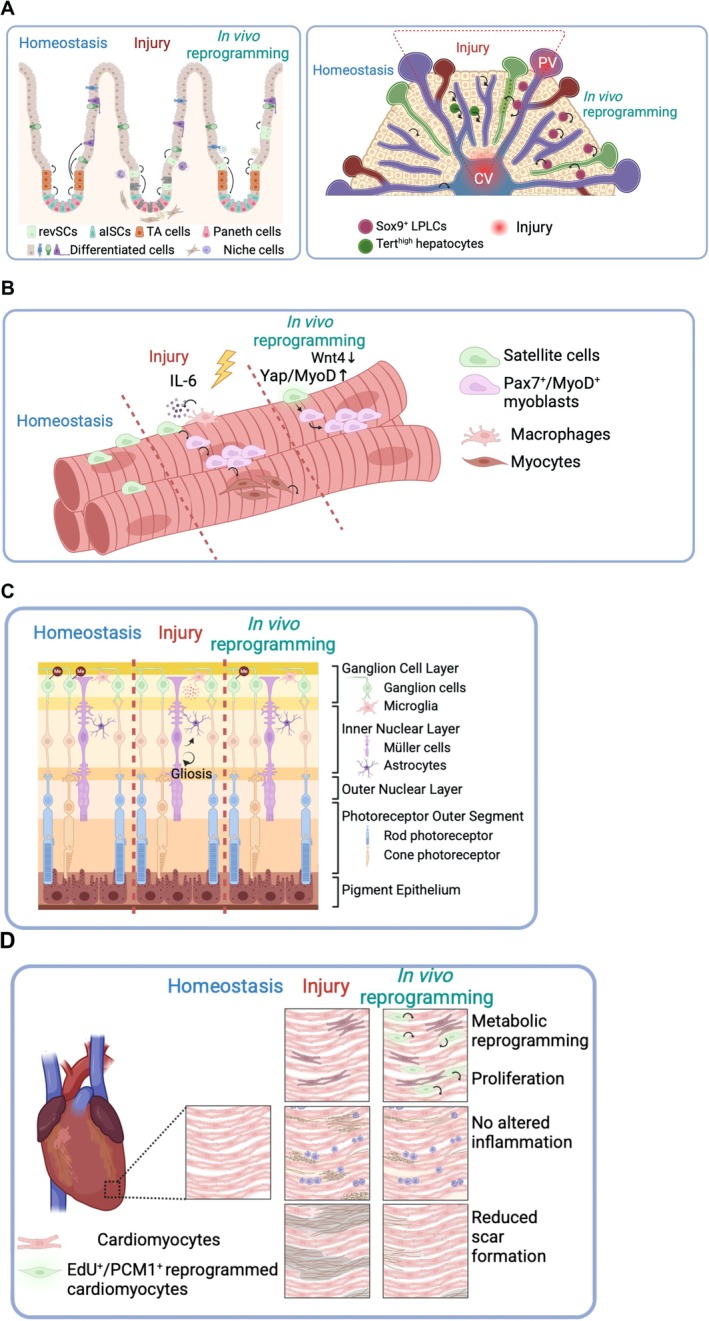
The shared regenerative mechanism of OSKM reprogramming and injury repair in multiple organs. Each organ is depicted in three distinct stages: Homeostasis (blue), injury (red), and in vivo reprogramming (green). (A) Intestine (left panel): As a regenerative organ with resident active intestinal stem cells (aISCs), the intestinal epithelium maintains homeostasis by continuously generating transit‐amplifying (TA) cells and various differentiated cell types. Upon injury, which eliminates aISCs, multiple epithelial cell types, including differentiated cells, undergo dedifferentiation to generate transient regenerative progenitors, termed revival stem cells (revSCs). This process is mediated by the secretion of prostaglandins from niche cells such as fibroblasts and macrophages. Under in vivo reprogramming, repair stem cell regeneration is induced autonomously, as epithelial cells themselves begin producing prostaglandins, facilitating intestinal tissue regeneration. Liver (right panel): As a highly regenerative organ that lacks resident stem cells, liver regeneration is primarily driven by hepatocyte proliferation and concomitant hepatic dedifferentiation. Upon injury, hepatocytes undergo dedifferentiation into Sox9+ liver progenitor cells, a process mediated by IL‐6 secretion from active Kupffer cells. These progenitor‐like cells contribute to the generation of new hepatocytes, restoring liver function. In the context of in vivo reprogramming, Sox9+ liver progenitor‐like cells are further induced to generate hepatocytes, thereby facilitating liver regeneration. (B) Skeletal muscle: As a regenerative organ, skeletal muscle contains resident quiescent stem cells such as PAX7^+^ muscle stem cells (i.e., satellite cells), which are essential for maintaining muscle integrity. Upon injury, these satellite cells become activated, leading to proliferation of PAX7+/MYOD+ myoblasts that subsequently differentiate and fuse to form new myofibers, facilitating muscle regeneration. In the context of in vivo reprogramming, satellite cell activation and proliferation are further enhanced through the activation of MYOD and YAP, a process facilitated by repression of *Wnt4* in myofibers. This modulation promotes efficient muscle regeneration by supporting the expansion and differentiation of satellite cells. (C) Retina: The retina is a non‐regenerative organ, and no resident stem cells have been identified. Upon injury, activated Müller cells undergo hypertrophy and exhibit reactive changes, a process known as gliosis. However, this response has limited regenerative potential due to Müller cells being highly restricted in proliferative ability. In the context of in vivo reprogramming, retinal regeneration is facilitated through TET2‐dependent DNA demethylation in retinal ganglion cells (RGCs), which promotes cellular plasticity and enhancing functional recovery. (D) Heart: The heart is a non‐regenerative organ that lacks resident stem cells. Cardiomyocytes exhibit very low epigenetic plasticity (or low reprogramming competence), leading to extremely limited regenerative capacity. In the context of in vivo reprogramming, high and prolonged expression of OSKM is required. The reprogramming process is accompanied by active cell cycle reentry in cardiomyocytes, along with metabolic changes that promote cardiac functional improvement and reduce fibrosis, enhancing overall cardiac regeneration.

In the liver, emerging evidence suggests OSKM to downregulate hepatocyte identity genes while activating fetal programs, paralleling injury‐induced LPLC formation but occurring independently of cytokine inputs (unpublished observations) (Figure 4A). In skeletal muscle, OSKM enhances regenerative capacity by expanding PAX7^+^ satellite cells (Figure 4B), whereas in non‐regenerative organs such as the retina (Figure 4C), which lack endogenous dedifferentiation, OSKM may instead act via epigenetic rejuvenation, restoring function without producing TRPs. In the adult mammalian heart, injury elicits only partial dedifferentiation (sarcomere disassembly, fetal gene reactivation) (Yao and Wang 2020; Zhu et al. 2021), whereas zebrafish cardiomyocytes undergo robust dedifferentiation and regeneration (Jopling et al. 2010; Kikuchi et al. 2010). OSKM appears to stabilize and amplify these incomplete processes, shifting outcomes from fibrosis toward functional repair (Chen et al. 2021).

Together, these observations suggest that reprogramming competence—the intrinsic ability of a given cell type to undergo chromatin remodeling and adopt regenerative plasticity (Li et al. 2020)—determines whether OSKM induction yields TRP‐like intermediates (as in the intestine and liver) or instead promotes functional rejuvenation (as in the retina and heart). In this way, OSKM reprogramming both recapitulates and extends injury‐induced dedifferentiation, providing a powerful tool to unlock regenerative potential across tissues with differing intrinsic capacity.

## 4 Rejuvenation by In Vivo Reprogramming

### 4.1 OSKM Reprogramming and Epigenetic Remodeling

A pioneering study by the Belmonte group revealed that in vivo OSKM induction can promote rejuvenation in mouse models carrying the *Lmna* G609G mutation, which recapitulates HGPS (Ocampo et al. 2016). This was surprising, as complete reprogramming of HGPS fibroblasts into iPSCs eliminates progerin expression entirely (Liu et al. 2011) and restores normal nuclear morphology (Chen et al. 2017). By contrast, partial reprogramming by OSKM does not reduce progerin level (Ocampo et al. 2016), but instead alleviates disease phenotypes through epigenetic remodeling. Specifically, OSKM restores heterochromatin marks disrupted in HGPS and physiological aging, including reduced H3K27me3 and H3K9me3 (Shumaker et al. 2006) and elevated H4K20me3 (Liu et al. 2013), which are otherwise linked to persistent DNA damage (Benayoun et al. 2015; Liu et al. 2013). These findings suggest that OSKM reprogramming reverses aging‐associated epigenetic drift, even without altering the causative protein (progerin).

A key advance in this field was the development of the Inducible Changes to the Epigenome (ICE) mouse model, wherein double‐strand DNA breaks are introduced exclusively in non‐coding regions, thereby avoiding mutations in protein‐coding genes while still triggering a DNA damage response (Yang, Hayano, et al. 2023). Despite the absence of coding mutations, ICE mice exhibit accelerated hallmarks of aging, including epigenetic erosion (loss of H3K27ac), disrupted cell identity, and impaired tissue function. These findings support the Information Theory of Aging, which posits that aging arises from progressive loss of epigenetic information rather than accumulation of mutations in coding regions (Lu et al. 2023). Importantly, transient OSK expression in the retina via viral delivery or transgenic expression locally reversed these epigenetic defects and restored function, demonstrating that OSKM‐mediated reprogramming can reset the epigenetic landscape, reverse aging‐associated decline, and rescue function (Parras et al. 2023; Yang, Hayano, et al. 2023).

Despite these promising benefits, the risks associated with continuous OSKM expression are considerable. Sustained induction can cause tumor formation (Ohnishi et al. 2014), teratoma development (Abad et al. 2013), and even premature lethality due to intestinal or liver failure (Parras et al. 2023). To address this, a cyclic induction protocol (e.g., 2 days ON and 5 days OFF per week) has been developed (Browder et al. 2022). This approach mitigates the weight loss and mortality observed with continuous expression (Ocampo et al. 2016) while still promoting rejuvenation and hence represents a more feasible path for long‐term application.

### 4.2 Systemic Effects of OSKM Reprogramming on Longevity

In physiologically aged mice, long‐term cyclic induction of OSKM restores youthful multi‐omics signatures—including DNA methylation, transcriptomic, and lipidomic profiles—across multiple organs such as the spleen, liver, skin, kidney, lung, and skeletal muscle (Browder et al. 2022). Importantly, this regimen also promotes functional regeneration: while short‐term reprogramming enhances muscle repair through local niche control (Wang et al. 2021), sustained cyclic reprogramming improves wound healing and reduces fibrosis in both muscle and skin (Browder et al. 2022). Consistent with these findings, the Serrano group showed that even a single 1‐week cycle of OSKM in aged mice (55 weeks) elicits systemic rejuvenation, evidenced by DNA methylation reprogramming across the pancreas, liver, spleen, and blood (Chondronasiou et al. 2022).

Schwann cells of the peripheral nervous system undergo adaptive cellular reprogramming, or injury‐induced dedifferentiation, to achieve plasticity after injury (Jessen et al. 2015). In this state, they transition into repair cells that drive axonal regrowth and remyelination, while also stimulating progenitor proliferation to rebuild damaged tissue (Carr and Johnston 2017). In contrast, most neuronal cells in the central nervous system have long been considered refractory to regeneration due to the post‐mitotic nature of neurons and the limited proliferative capacity of glia (Doetsch et al. 1999). Nonetheless, accumulating evidence shows that partial OSKM reprogramming can reverse age‐related decline in the brain, primarily through epigenetic restoration rather than dedifferentiation. In the aged dentate gyrus, cyclic induction (e.g., 3 days ON/4 days OFF over 4–6 months) reduces lipofuscin accumulation, restores heterochromatin integrity (H3K9me3), decreases DNA damage (γH2AX), and reinstates a youthful molecular profile, accompanied by improved memory (Rodriguez‐Matellan et al. 2020). Similar long‐term cyclic induction protocols in Alzheimer's disease models restore synaptic integrity, reduce amyloid burden, and normalize mitochondrial and proteostatic homeostasis without inducing gliosis or loss of neuronal identity (Shen et al. 2024). Complementary studies further demonstrate that systemic cyclic induction for three cycles, as well as region‐specific OSKM induction for 7 days by stereotaxic AAV‐Cre delivery in the subventricular zone, enhance neuroblast differentiation and reverse age‐related transcriptional signatures while preserving lineage fidelity (Xu et al. 2024). In neuron‐restricted models, cyclic induction improves hippocampal activity, restores youthful heterochromatin (H4K20me3), and enhances memory performance, whereas continuous expression is ineffective or deleterious (Anton‐Fernandez et al. 2024). Notably, the age‐associated loss of Schwann cell plasticity can also be restored by partial OSKM induction, which resets stress granule homeostasis, corrects dysfunctional Runx2+ transitional states, and thereby enables efficient axon regeneration in aged nerves (Wang, Wang, et al. 2025).

However, not all systems show rejuvenation benefits with OSKM induction. In *Caenorhabditis elegans* , neuron‐specific overexpression of OSK orthologs failed to improve short‐term memory or extend lifespan; in fact, it disrupted chemotaxis behaviors, suggesting possible deleterious effects on postmitotic neurons (Toraason et al. 2024). These findings underscore that the efficacy of partial reprogramming may depend on species‐ and cell‐type–specific mechanisms such as DNA‐methylation–based epigenetic regulation, which is absent in nematodes but central to mammalian aging.

### 4.3 Translational Potential and Safety Considerations for Human Applications

As a feasible strategy for use in humans, consecutive delivery of a mRNA cocktail of OSKM plus *LIN28* (L) and *NANOG* (N) (OSKMLN), optimized for transgene‐free full reprogramming (Yusa et al. 2009), for 4 days prior to the “Point of No Return” (Nagy and Nagy 2010) has been demonstrated to ameliorate cellular aging through partial OSKM reprogramming (Sarkar et al. 2020). Rejuvenating effects of reprogramming have been demonstrated in somatic cells from aged human fibroblasts, endothelial cells, and diseased chondrocytes, and functionally validated in aged muscle stem cells from mice (Sarkar et al. 2020); in addition, the effects of OSKM reprogramming in human cells and mouse models have been further characterized by a number of studies (Gill et al. 2022; Olova et al. 2019; Rodriguez‐Matellan et al. 2020; Roux et al. 2022), with several review articles summarizing the current state of this growing field (Cipriano et al. 2024; Paine et al. 2024; Puri and Wagner 2023; Sichani et al. 2024; Yucel and Gladyshev 2024; Zhang et al. 2020). However, a few safety concerns should be considered with regard to systemic application, such as the potential risks of tumor formation and loss of cell identity due to untimely OSKM reprogramming (Roux et al. 2022). The Belmonte group successfully achieved targeted OSKM reprogramming specifically in age‐associated cells by driving OSK gene expression under the promoter of *Cdkn2a*, a key gene involved in senescence induction; remarkably, both the HGPS mouse model and naturally aged mice demonstrated extended lifespans and delayed aging phenotypes without tumor formation (Sahu et al. 2024).

## 5 Premature Organ Failure With in Vivo Reprogramming

Formation of teratomas, a hallmark of pluripotent stem cells (PSCs), has been considered a major risk for stem cell therapies derived from human PSCs (Jeong et al. 2017). Accordingly, teratoma and/or tumor formation following full (Abad et al. 2013) or premature (Ohnishi et al. 2014) reprogramming is an anticipated risk of systemic OSKM induction. Beyond such tumorigenesis, studies in mice have shown continuous OSKM induction to potentially trigger rapid “sickness,” manifested as weight loss, reduced activity, and mortality in as little as 4 days, before teratomas develop (Ocampo et al. 2016). Subsequent studies have identified intestinal and liver failure as the primary cause of this lethality, with mortality varying by genetic background and OSKM cassette locus (higher in *Col1a1*‐driven 4Fj than in *Pparg*‐driven 4Fs‐B mice, correlating with elevated OCT4 expression in gut and liver) (Parras et al. 2023). Similar systemic toxicity was confirmed in *C. elegans* with inducible OSKM expression (Kamaludeen et al. 2024).

To mitigate these effects, a “cyclic induction protocol” (e.g., 2 days ON, 5 days OFF per week) has been established that allows safe long‐term reprogramming for up to 35 cycles (Ocampo et al. 2016) or 10 months (Browder et al. 2022). In addition, mortality was further reduced by restricting OSKM expression in gut and liver, either through tissue‐specific Cre drivers or engineering of chimeric strains (Pico et al. 2025). Together, these findings underscore the need for strategies that avoid hepatic and intestinal toxicity in translational applications. Promising alternatives include local OSK delivery via AAV vectors (Lu et al. 2020; Yang, Hayano, et al. 2023) or ex vivo transplantation of transiently reprogrammed stem cells, which enable organ‐specific regeneration without systemic toxicity. Meanwhile, realizing systemic rejuvenation without prolonged OSK expression remains challenging. AAV9‐mediated OSK delivery in aged mice extended lifespan and reversed aging phenotypes (Macip et al. 2024), but still‐safer approaches may be required. One promising approach is to use small‐molecule cocktails originally developed for chemical reprogramming (Hou et al. 2013), some of which have successfully reversed senescence in human fibroblasts (Yang, Petty, et al. 2023), achieved full iPSC reprogramming (Guan et al. 2022; Liuyang et al. 2023; Wang, Peng, et al. 2025), or extended lifespan in *C. elegans* (Schoenfeldt et al. 2022), supporting their potential as a translatable alternative for systemic rejuvenation.

## 6 Prolonged Epigenetic Reprogramming for Rejuvenation

OSKM reprogramming for injury regeneration can be achieved through short‐term induction (e.g., as little as 3 days), whereas epigenetic reprogramming for rejuvenation requires a significantly longer duration (e.g., up to several months) (Browder et al. 2022). The loss of cell identity during OSKM reprogramming is well‐documented, as demonstrated by the marked reduction of intestinal secretory cells (Kim et al. 2023) and the effects observed in human dermal fibroblasts (Olova et al. 2019) following short‐term induction. This suggests that the epigenetic landscape regulating cell identity in highly reprogrammable cells—those with enhanced dedifferentiation potential or plasticity due to innate high reprogramming competence (Figure 3E)—is inherently more dynamic and adaptable than the more stable, age‐associated epigenetic modifications such as H4K20me3, H3K9me3, and H3K9ac (Sidler et al. 2017). For instance, H4K20me3, which is completely lost in senescent cells (O'Sullivan et al. 2010) and generally reduced in the aging mouse brain (Wang et al. 2010), has been shown to be restored in the brain following an 11‐month cyclic reprogramming protocol (Anton‐Fernandez et al. 2024). Similarly, the age‐related decline in H3K9me3 can be prevented through a 4‐month cyclic reprogramming regimen (Rodriguez‐Matellan et al. 2020). Transcriptomic analyses of in vitro reprogramming further highlight the temporal dynamics of OSKM‐mediated epigenetic remodeling. Two distinct transcriptional modules have emerged: an early‐induced cluster, enriched for proliferation and biosynthetic genes, and a late‐induced cluster, encompassing pluripotency regulators and chromatin remodelers (Buganim et al. 2012; Tanabe et al. 2013). These transcriptional waves align with stage‐specific epigenetic transitions: first the early loss of somatic lineage marks, followed by the late re‐establishment of bivalent domains (H3K4me3/H3K27me3) at lineage‐specific genes (Apostolou and Hochedlinger 2013). Importantly, iPSCs derived from Alzheimer's disease patient fibroblasts show only mild disease‐associated transcriptomic signatures once differentiated into neurons, whereas directly transdifferentiated neurons retain a strong pathological profile (Mertens et al. 2021). This underscores that sustained epigenetic reprogramming—sufficient to reset age‐associated chromatin states—is essential for erasing degenerative memory and offers a potential route for recovering tissue function in aging and neurodegeneration. Consistent with this, a recent meta‐analysis identified “mesenchymal drift”—the age‐associated shift of diverse cell types toward aberrant stromal and fibroblast‐like transcriptional programs—as a conserved hallmark of aging that can be reversed by partial OSKM reprogramming, reinforcing its systemic rejuvenation potential (Lu et al. 2025).

## 7 Concluding Remarks

In vivo reprogramming with OSK(M or MLN) has shown strong potential to restore youthful epigenetic profiles in aged cells and enhance reprogramming competence, thereby enabling the formation of TRPs across multiple organs and promoting both rejuvenation and tissue regeneration. Nevertheless, significant challenges to its application remain, including tumor formation, intestinal and liver failure, and loss of cellular identity. Achieving precise spatiotemporal control over reprogramming will be essential to minimize these risks while preserving therapeutic benefits. Future efforts should prioritize refining delivery methods and exploring safer alternatives such as small molecules or modified gene sets.

Interest in this field is rapidly growing within the biotech sector, as summarized in recent dedicated reviews (de Magalhaes and Ocampo 2022; Eisenstein 2022; Paine et al. 2024; Pereira et al. 2024), which provide detailed accounts of company pipelines and translational strategies. In this review, we instead focused on mechanistic insights into injury‐induced and OSKM‐induced reprogramming, offering a framework for understanding how regenerative competence can be harnessed across tissues. With careful modulation, OSKM‐based approaches hold strong potential to transform regenerative medicine and the treatment of age‐related diseases (Box 1).

BOX 1

**Table:** 

Term	Definition
Regeneration	Restoration of tissue structure and function following injury, typically involving cell proliferation, dedifferentiation, or activation of progenitor cells.
Rejuvenation	Molecular or epigenetic reversal of age‐associated changes without altering cell identity; often involves resetting gene expression or chromatin states.
Partial reprogramming	Transient OSKM expression that induces a youthful, plastic state without full pluripotency or activation of core pluripotency genes (e.g., *Nanog* and *Pou5f1*).
Full reprogramming or induced pluripotency	Sustained OSKM expression that activates endogenous pluripotency networks and leads to the acquisition of pluripotent potential, including teratoma formation.
Short‐term versus long‐term OSKM	Short‐term OSKM refers to induction over days to < 2 weeks (non‐pluripotent), while long‐term OSKM spans ≥ 2–4 weeks and risks full reprogramming and tumorigenesis.
Reprogramming competence	A cell's intrinsic ability to respond to OSKM and undergo dedifferentiation, proliferation, or epigenetic remodeling, depending on context and duration of induction.
Transient regenerative progenitors	Dedifferentiated cells that emerge in response to injury, acquire transient stem‐like properties, and contribute to tissue restoration; their induction is driven by extrinsic signals (e.g., inflammation, ECM remodeling, cytokines such as IL‐6, TGFβ, or PGE2) that reprogram mature parenchymal or epithelial cells into regenerative intermediates.
Mesenchymal drift	An age‐associated transcriptional shift in diverse cell types toward aberrant stromal and fibroblast‐like gene expression programs, contributing to tissue dysfunction and disease.

## Author Contributions

H.‐J.C. was involved in conceptualization, supervision, critical revisions, and funding acquisition. J.K. was involved in literature synthesis and interpretation, drafting, and overall revision. B.‐K.J. was involved in literature search, drafting, and review. S.‐Y.L. and H.‐J.E. were involved in literature search and editing. All authors read and approved the final manuscript.

## Conflicts of Interest

The authors declare no conflicts of interest.

## Data Availability

Data sharing not applicable to this article as no datasets were generated or analyzed during the current study.
